# Prediction of structural stability of short beta-hairpin peptides by molecular dynamics and knowledge-based potentials

**DOI:** 10.1186/1472-6807-8-27

**Published:** 2008-05-29

**Authors:** Karin Noy, Nir Kalisman, Chen Keasar

**Affiliations:** 1 Department of Life Sciences, Ben-Gurion University, Beer-Sheva, Israel; 2 Department of Computer Sciences, Ben-Gurion University, Beer-Sheva, Israel

## Abstract

**Background:**

The structural stability of peptides in solution strongly affects their binding affinities and specificities. Thus, in peptide biotechnology, an increase in the structural stability is often desirable. The present work combines two orthogonal computational techniques, Molecular Dynamics and a knowledge-based potential, for the prediction of structural stability of short peptides (< 20 residues) in solution.

**Results:**

We tested the new approach on four families of short β-hairpin peptides: TrpZip, MBH, bhpW and EPO, whose structural stabilities have been experimentally measured in previous studies. For all four families, both computational techniques show considerable correlation (r > 0.65) with the experimentally measured stabilities. The consensus of the two techniques shows higher correlation (r > 0.82).

**Conclusion:**

Our results suggest a prediction scheme that can be used to estimate the relative structural stability within a peptide family. We discuss the applicability of this predictive approach for in-silico screening of combinatorial peptide libraries.

## Background

Peptides are important constituents of biological systems. They often initiate signal transduction cascades by binding and activating membrane-bound receptors [1,2]. Many therapeutic peptides exert their activity by binding to these receptors, and either activating [3] or blocking them [4]. Another type of peptide therapeutic activity is the prevention of disease-related protein-protein interactions. In this case, peptides derived from one of the proteins compete against it over the interaction site [5].

In all these cases the key to the therapeutic effect is high affinity binding of the peptide to a specific site in the target protein. This, in turn, depends on the peptide's ability to adopt a binding-site-compatible conformation. The more stable this conformation is, the higher the affinity [6,7] due to the lower entropic price of binding. Structural stability of the bound conformation also affects the specificity of binding, because of the inverse correlation between stability and the accessibility to alternative binding conformations. Natural peptides are often rather flexible and long and may achieve specificity and affinity by binding to side-sites. Therapeutic peptide and peptide-like agents, on the other hand, must be kept short for pharmaceutical reasons, and their development often involves rigidification [6-8].

The search for specific high-affinity binding peptides requires the screening of a large number of candidates, often using combinatorial libraries [9,10]. Such libraries however carry a very high price tag, for both creation and screening. In other fields of combinatorial chemistry, this problem is partially alleviated by the screening of virtual libraries [11]. With peptides however, widespread application of virtual screening is hindered by the difficulty of peptide structure prediction [12], and the absence of reliable methods for the prediction of their structural stability. In some highly constrained cases, most notably MHC binding peptides, these issues were partially solved, and useful virtual screening was achieved [13]. This however is not the general rule. Thus, the difficulty of computational prediction schemes for structure and structural stability of peptides is a major obstacle to virtual screening of peptides, and to full realization of the therapeutic potential of peptides.

As with proteins, the problems of predicting peptide structure and structural stabilities are challenging. The free energy differences between the folded and unfolded states are marginal compared with the accuracy of the current computational tools. In the field of protein structure prediction, homology modeling circumvents much of the structure prediction problem. Similarly, one may synthesize a single peptide, determine its structure experimentally and assume that this structure is shared by a large number peptides with a similar sequence [14-19]. However, since minor dissimilarity in sequence may result in considerable difference in the stability, the problem of structure stability prediction is more difficult to circumvent.

The current work aims to cope with the structural stability problem using a two-fold strategy. First, instead of predicting absolute stabilities we focus on predicting relative stabilities within a family of similar peptides. Second, we use two complementary tools: Molecular Dynamics (MD) and a Knowledge-Based Potential (KBP).

MD is a common technique for structural studies of proteins and peptides [24-26]. It uses accurate semi-empirical forcefields, and is able to reproduce equilibrium, entropy-based phenomena by substantial sampling of the conformational space [27-29]. Its major disadvantage is that very long simulations are required to reach equilibrium. In a series of pioneering studies Zanuy *et al*. [20-22] and Tsai *et al*. [23] used Molecular Dynamics simulations to compare the relative stabilities of different possible configurations of amyloid peptides. Their results, however, were not directly correlated with experimentally measured stabilities. Furthermore, the MD protocols they used are too computationally intensive to allow large-scale stability prediction projects.

Fortunately, much insight can be gained from non-equilibrium simulations that may be relatively short. Specifically, unfolding MD simulations of proteins qualitatively reproduce the unfolding pathways. Those structural features that seem most stable in unfolding experiments, also persist (on the average) longer in the simulations [30,31]. Our working assumption was that a similar trend would appear in unfolding simulations of peptides, so that more stable peptides would, on average, retain their structure longer than non-stable ones.

The alternative complementary approach we use is based on a backbone conformation KBP. Experimental and computational evidences confirm the essential role of local residue preferences in shaping protein structures [32-35]. These studies motivated several KBPs that scored the compatibility of short fragments in a protein with a given conformation, and were used mainly to sort out native structures from non-native decoys [34-36]. Our working hypothesis was that in peptides the innate preferences of the residues would exert an even stronger effect than in proteins, since peptides are too small to support a considerable hydrophobic core. Thus, a KBP that measures the compatibility of peptide conformations with the innate backbone preferences of the residues may estimate structural stability. The KBP presented here is similar in spirit to previously published ones [37,38]. We use it to estimate the likelihood of finding the residues of a peptide in specific {*Phi, Psi*} configurations.

**Figure 1:**
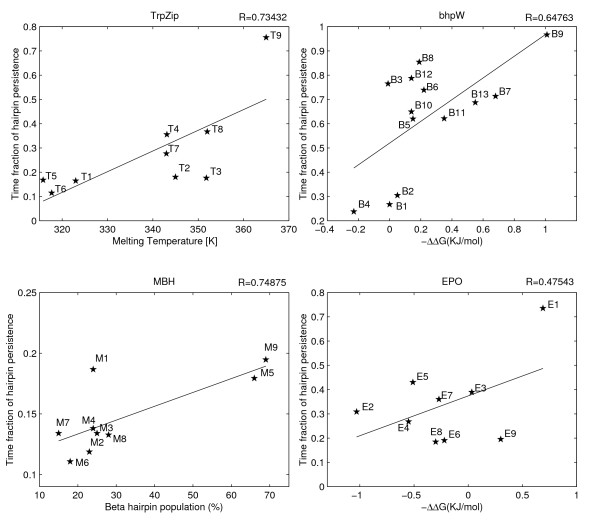
**The correlations between the computed and the experimentally measured stabilities**. The structural stabilities during MD simulations (Y-axis) are plotted against the experimentally measured stabilities of TrpZip, bhpW, MBH and EPO peptides (X-axis). The structural stability of EPO and bhpW was measured by effective concentration of peptide thiols in -ΔΔG units [17–19]; the structural stability of MBH/BHKE was measured by NMR in population [%] units [14–15]; and the structural stability of TrpZip was measured by CD in Tm[K] units [16]. The structural stability during MD was measured by the fraction of time in which the RMSD_all-atoms _is below 2.6Å. Correlation coefficients are indicated. The p-values of the correlation coefficients are 0.2, 0.02, 0.016, 0.02 for EPO, TrpZip, bhp, and MBH respectively.

**Figure 2:**
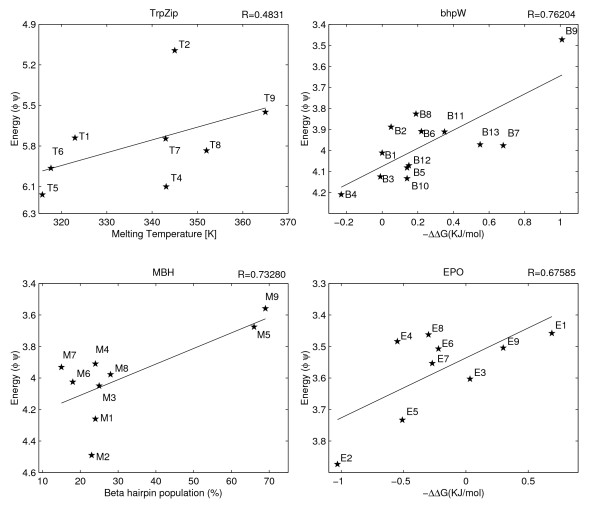
**The correlations between the KBP and the experimentally measured stabilities**. The peptide KBP energies (Y-axis) are plotted against the experimentally measured stabilities of TrpZip, bhpW, MBH and EPO peptides (X-axis). The KBP has arbitrary energy units. Correlation coefficients are indicated. The p-values of the correlation coefficients are 0.045, 0.22, 0.002, 0.025 for EPO, TrpZip, bhp, and MBH respectively.

**Figure 3:**
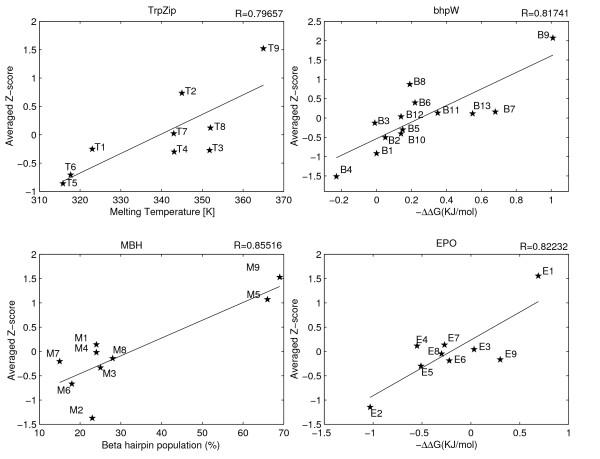
**The correlations between the average Z-score of the two computational techniques and the experimental stabilities**. The averaged Z-scores (Y-axis) are plotted against the experimentally measured stabilities of TrpZip, bhpW, MBH and EPO peptides (X-axis). Correlation coefficients are indicated. The p-values of the correlation coefficients are 0.0065, 0.01, 0.006, 0.003 for EPO, TrpZip, bhp, and MBH respectively.

Our structural stability prediction scheme requires structural models of the peptides. Unfortunately, due to the difficulty of peptide structure determination, experimentally based structures are scarce. Only 8 out of the 40 peptides used in this study have a known structure (Table 1) and similar or worse proportion are likely in any real-life scenario. Thus, we need to make do with the second best option, template-based modelling. The unknown accuracy of this modeling adds to the noise in our prediction. This however, should not be a major source of errors since we neither try to predict the most stable conformations of the peptides nor the stabilities of the most stable conformations. We try to estimate the stability in a predefined conformation (i.e., the conformation of the template) and if a peptide is uncomfortable in that conformation we assume it will be instable. It should be noted that this approach is compatible with the design of the experimental work that we try to model. The experiments measure the stability of a beta hairpin conformation and not the stabilities of other conformations which may exist or even be dominant. The EPO4 (E2 in Figures 1, 2, 3) peptide demonstrates this point [19]. While an NMR study indicates that it has a stable alpha-helix conformation, it is reported as non-stable (i.e., having positive ΔΔG) by the effective concentration of the peptide thiols, which estimate the stability of cysteine terminated beta-hairpins.

**Table 1:** The 40 peptides studied in this work

*Peptide Name*^A^	***Peptide ID***^B^	**Peptide Sequence**^C^	**Template (PDB)**^D^	**Substitutions**^E^
**TrpZip**				
TrpZip1	T1	S-**W**T**W**-EGNK-**W**T**W**-K	1LE0	None
TrpZip2	T2	S-**W**T**W**-ENGK-**W**T**W**-K	1LE1	None
TrpZip3	T3	S-**W**T**W**-EpNK-**W**T**W**-K	1LE0	G6→D-proline
TrpZip4	T4	GE**W**T**WDDATKTW**T**W**TE	1LE3	None
TrpZip5	T5	GE**W**TY**DDATKT**FT**W**TE	1LE3	W5→ Y
				W12→ F
TrpZip6	T6	GE**W**T**WDDATKTW**TVTE	1LE3	W14→V
TrpZip7	T7	GE**W**V**WDDATKTW**H**W**TE	1LE3	T4→ V
				T13→H
TrpZip8	T8	GE**W**H**WDDATKTW**V**W**TE	1LE3	T4→H
				T13→V
TrpZip9	T9	GE**W**V**WDDATKTW**V**W**TE	1LE3	T4→V
				T13→V
**BhpW**				
TT	B1	**C**T**WEGNKL**T**C**	1N09	None
HT	B2	**C**H**WEGNKL**T**C**	1N09	T2→H
TH	B3	**C**T**WEGNKL**H**C**	1N09	T9→H
LT	B4	**C**L**WEGNKL**T**C**	1N09	T2→L
TL	B5	**C**T**WEGNKL**L**C**	1N09	T9→L
VT	B6	**C**V**WEGNKL**T**C**	1N09	T2→V
TV	B7	**C**T**WEGNKL**V**C**	1N09	T9→V
VH	B8	**C**V**WEGNKL**H**C**	1N0D	None
HV	B9	**C**H**WEGNKL**V**C**	1N0C	None
TW	B10	**C**T**WEGNKL**W**C**	1N09	T9→W
FT	B11	**C**W**FEGNKL**T**C**	1N09	T2→ W
				W3→F
TF	B12	**C**T**WEGNKL**F**C**	1N09	T9→F
TI	B13	**C**T**WEGNKL**I**C**	1N09	T9→I
				T13→V
**BHKE/MBH**				
BHKE	M1	**RGK**ITV**NG**KTY**EGR**	1J4M	W4→ I
				Y6→V
				I9→K
MBH6	M2	**RGK**WTP**NG**HTD**EGR**		Y6→ P
				I9→H
MBH8	M3	**RGK**WTY**NG**HTD**EGR**		I9→H
				Y11→D
				I9→H
MBH10	M4	**RGK**WTD**NG**ITY**EGR**		Y6→D
MBH12	M5	**RGK**WTY**NG**ITY**EGR**		None
MBH20	M6	**RGK**YTP**NG**ITD**EGR**		W4→Y
				Y6→ P
				Y11→D
MBH21	M7	**RGK**YTY**NG**ITD**EGR**		W4→Y
				Y11→D
MBH28	M8	**RGK**YTD**NG**ITY**EGR**		W4→Y
				Y6→D
MBH36	M9	RGKYTYNGNTYEGR		Y6→D
				I9→N
**EPO**				
EPO3	E1	**SC**HFG**PLGW**V**CK**	1KVG	None
EPO4	E2	**SC**RAQ**PLGW**L**CK**		H3→R
				F4→ A
EPO8	E3	**SC**HFG**PLGW**L**CK**		V10→L
EPO9	E4	**SC**RAG**PLGW**L**CK**		V10→L
				H3→ R
				F4→A
EPO11	E5	**SC**HAG**PLGW**L**CK**		F4→A
				V10→L
EPO12	E6	**SC**RFG**PLGW**L**CK**		H3→ R
				V10→L
EPO14	E7	**SC**HAG**PLGW**V**CK**		F4→A
EPO16	E8	**SC**RAG**PLGW**V**CK**		H3→R
				F4→A
EPO21	E9	**SC**RFG**PLGW**V**CK**		H3→R

**Figure 4:**
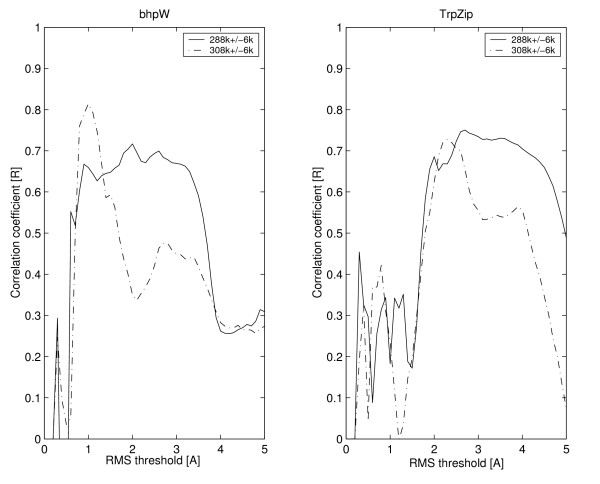
**Sensitivity of the MD based stability estimate to the temperature and RMSDall-atoms threshold**. The correlations between the median structure persistencies and the experimentally measured stabilities are plotted against the threshold at two temperatures (288°K and 308°K) and for two peptide families (TrpZip and bhpW). The lower temperature was selected for further analysis as it shows higher and more stable correlation. The chosen threshold, 2.6Å, is in the middle of an almost constant correlation region.

**Figure 5:**
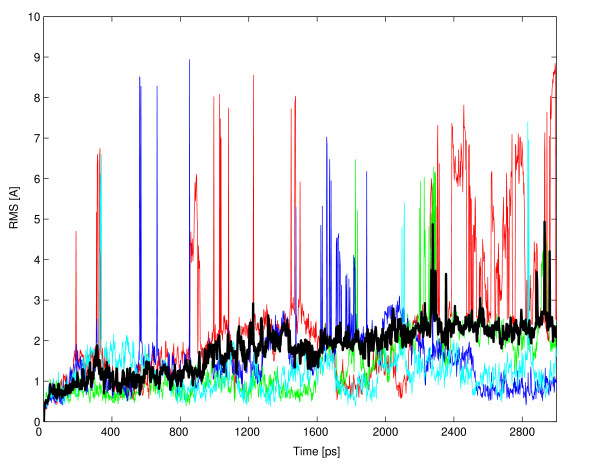
**Four trajectory simulations of the peptide bhp HV**. The root mean square deviation of all the peptide atoms from the initial structure (RMSDall-atoms) is plotted over time for four representative simulation runs. Six more runs were performed but their trajectories were omitted for clarity. The black line represents the median RMSDall-atoms of all ten trajectories.

Columns are as follows: A – Peptide name in the literature [14-19]. B – Peptide ID in figures 3-5. C – Amino acid sequence. Bold letters indicate invariant residues, p = D-proline. D – Protein Data Bank (PDB) [50] code of the NMR structures used as a template. For each NMR ensemble the first structure was employed. E. The amino acid substitutions applied on the templates in order to prepare the peptide models.

## Results

### Preliminary MD simulations

In this work we use the persistence of beta-hairpin conformations during MD simulations as an estimate for the structural stabilities of peptides. We measure this persistence by the fraction of the simulation time in which the median RMSD_all-atoms _(see Methods section for definition) of the peptide fall below some threshold. This fraction obviously depends not only on the innate properties of the peptide itself, but also on the chosen threshold and the MD parameters. Therefore, it is important to understand the dependencies between these parameters, if adequate correlation to the experimental stabilities is sought. The most important MD parameter is the simulation temperature. Under low temperature all peptides keep their structure, with only limited and hard-to-interpret perturbations. Under high temperature all peptides lose their structure almost instantaneously, again providing very little insight (data not shown). At two of the temperatures that we checked, 288°K and 308°K, all the peptides studied are marginally stable. That is, they keep their initial structure for some time along the simulation and then unfold at some point. For both temperatures, we tested the correlation between the persistence of the beta-structure and the experimentally measured stability in the bhpW and TrpZip families over a wide range of threshold values (Figure 4). In the 308°K simulations this correlation is very sensitive to the exact threshold value and thus seems to be an unreliable predictor. In the 288°K runs, on the other hand, the correlation is almost constant for both families within the threshold range of 1.5–3.5Å. In the following sections, we report only the results for 288 K simulations and a threshold of 2.6Å (the middle of this range).

### Considerable correlation between computed and experimentally measured structural stabilities

Figures 1 and 2 show the correlation between the computed and the experimental stabilities for each of the four peptide-families and for each computational technique. The peptide families were studied with different experimental methods, and their stabilities were reported in different units. These differences, however, do not affect the analysis, as we do not compare the stabilities of peptides across families. By restricting our comparison to peptides within the same family, we also discard the concern about different peptide lengths between the different families, which influences both computational techniques.

The MD technique (Figure 1) shows an average correlation of 0.65 ± 0.12 (average ± SD) between the computed and the experimental stabilities. The technique was also able to consistently point to the most stable peptide in each family. The best predicted stabilities, with a correlation coefficient larger than 0.7, are obtained for the TrpZip and MBH peptide families. The prediction for the EPO family was the least accurate and the only statistically insignificant one. The persistence fraction of time, in which a peptide is within 2.6Å of its initial conformation, varies considerably between the families, averaging 60% in bhpW and less than 20% in MBH. In contrast to this inter-family heterogeneity, the ratios of persistence times between the most and the least stable peptide within the same family is 2–3 folds in all families.

The correlations between the KBP and peptide stabilities (Figure 2) have a negative sign as higher energies imply lower stabilities. Their average magnitude, however, is a bit higher than that of the MD correlations, 0.66 ± 0.12. The application of this technique is slightly more restricted than MD, because the KBP is only defined for naturally occurring amino acid types. Consequently, the TrpZip3 peptide, which contains a D-amino acid (D-Proline) in its chain, had to be eliminated from this data set. The peptide families with high correlation coefficients according to this technique are quite different than the ones found by MD. The KBP provides the best predicted stabilities (R < -0.7) for the bhpW and MBH peptide families. On the other hand, statistically insignificant prediction is obtained in the TrpZip family that had high correlation in the MD technique.

The overall performances of the two techniques are very similar, as the magnitudes of the average correlation coefficients are around 0.66 for both methods. The prediction details, however, are rather different. First, the accuracy of prediction for a certain peptide family might be higher with one method and lower with the other. Moreover, the stability of a peptide within a certain family may be over-predicted with one technique and under-predicted with the other. For example the EPO12 peptide (E6 in Figures 1-3) is predicted to be the second least stable of its family by MD and among the 4^th ^most stable by the KBP. These observations, as well as the different theoretical foundations of the two approaches, suggest that better correlation may be achieved by merging the predictions of the two techniques. Direct summation of both results is impossible because of unit discrepancy. The KBP stability estimator is an energy value in some arbitrary units, while the MD estimator is the fraction of time the peptide spent in the beta-hairpin conformation. To achieve comparable scales for both techniques we converted each stability estimate into its corresponding Z-score relative to the other peptide estimates from the same family. The TrpZip3 peptide that had no KBP estimation, because it includes the non-standard residue D-Proline, was arbitrarily assigned with the Z-score of 0. The final combined estimator for each peptide was simply the average of the MD Z-score and the negative value of the KBP Z-score (because of the negative correlation coefficient).

Indeed, the combined stability estimator shows considerably better correlation with the experimental results than any of the two techniques alone (Figure 3). The average correlation coefficient rises to r = 0.82 ± 0.02, and the correlations are all statistically significant (p ≤ 0.01) and within the narrow range of 0.8–0.85. Furthermore, bootstrapping suggests that these correlation values are rather robust. Subsets of each peptide family have similar median correlations and only slightly lower average correlations and 79% or more than of them are statistically significant (p < 0.05) (Table 2). The small variability in the four correlation values provided by the combined estimator is very different from the variable results obtained by the single technique estimates. As in the MD estimator case, the combined estimator is able to consistently point to the most stable peptide in each family, and in 3 out of the 4 cases also to the least stable peptide.

**Table 2:** Bootstrap Analysis

*family*	*All peptides*	*Bootstrapping*			
	
	*mean (Figure 3)*	*mean*	*median*	*standard deviation*	*% significant * *correlation* *coefficients*
bhpW	0.817	0.78	0.83	0.16	90%
TrpZip	0.797	0.78	0.80	0.15	84%
MBH	0.855	0.81	0.87	0.23	89%
EPO	0.822	0.73	0.82	0.30	79%

The mean, median and standard deviation of the correlation coefficients of 1000 bootstrap samples of each peptide family. The percentages of the statistically significant correlation coefficients (p-value < 0.05) are indicated in the last column. The correlation coefficients for the whole families (Figure 3) were added for completeness.

## Discussion and Conclusion

The structural stability of peptides profoundly affects their efficiency as therapeutic agents. Despite this, experimental quantitative data on peptide structural stability is scarce and computational studies are (to the best of our knowledge) non-existent. The current work is the first attempt to fill this void, which hampers wider use of virtual peptide libraries and screening. We use two orthogonal computational schemes to estimate the relative structural stabilities within peptide families. The KBP scheme estimates the local preferences of the residues in a peptide to adopt a conformation, based on database statistics. This scheme shows a -0.66 ± 0.12 average correlation with experimentally determined stabilities of peptides from four families. The MD scheme simulates the physical process of peptide unfolding in an explicit solvent, and shows a very similar average correlation with the experimental results (0.66 ± 0.12).

Each of the schemes has its own advantages over the other. The KBP calculations are practically instantaneous, while MD runs require hours of CPU time at the very least. In addition, the KBP may represent some aspects of local conformational preferences better than MD [47]. On the other hand, MD in an explicit solvent is likely to account better for the entropic effects of solvation. MD is also able to treat uncommon residue types (e.g. the D-Proline in TrpZip3), whereas the KBP cannot. As the two schemes are based on very different theoretical foundations, and because their advantages are complementary in many ways, their errors need not be correlated. A combined prediction approach, using a simple average of the two estimators, indeed showed a considerable improvement in the correlation with experimental results (0.82 ± 0.02).

Two previous studies compared KBPs with a detailed atomic potential [48] and with the results of MD simulations [49]. Both emphasized the correlation between the results of these theoretically unrelated approaches. We also observe this correlation, as the results of both KBP and MD correlate with the experimental results, and thus with one another. Our work however, is the first to take advantage of the low correlation between the errors of these approaches.

The major problem to any computational study in this field is the scarceness of experimental data, which raises two concerns: unstable results and overfitting. The bootstrapping analysis however, suggests that the results presented here are robust (Table 2). We tried to avoid the overfitting problem, at least partially, by using a minimal set of adjustable parameters. An immediate consequence of this requirement is our decision to focus on only one KBP. In principle, other terms like torsion-angle propensity [35] or solvation [51,52] might have added more information but at the same time their weights in the overall scheme would have been hard to learn without overfitting. In our scheme the only four user-defined parameters are the temperature of the MD simulation (288°K), the similarity threshold to the initial conformation (2.6Å), the length of the MD run (3000 ps) and the weights of the MD and KBP results in the averaged prediction. The length of the simulation was a direct consequence of the available computational resources. We simply used the longest runs we could afford. However, inspection of selected traces however, indicates that the exact length is not very influential (Figure 5). Of the other two MD parameters, the temperature is the more sensitive one. In fact, its selection was done with the aim of making the second MD parameter (the similarity threshold) as robust as possible (Figure 4). Finally, no attempt was made to optimize the combined estimator by differentially weighting the two techniques in the Z-score averaging. Figure 1 suggests that indeed no significant over-fitting occurred in the choice of the MD parameters. The performance over the two peptide families that were used in tuning the temperature and threshold (TrpZip and bhpW) is similar to the performance over the two other families.

These results suggest, for the first time, a rational strategy for virtual screening of potentially therapeutic peptides. Given a lead peptide with some weak desired activity and a known structure, a large number of similar peptides can be constructed and screened *in-silico*. First, their structural models will be built based on the assumed functional conformation of the lead, and then their relative stabilities, in that conformation can be estimated by the combined MD/KBP approach presented in this study. The peptides predicted to be most stable may then be synthesized and tested experimentally for enhanced affinity and specificity.

If the lead peptide is a competitive inhibitor of an interaction between its protein of origin and another protein, and if the structure of the protein complex is known, a similar screening scheme may be applied even in the absence of an experimental structure of the lead, which is often unstructured when unbound. The initial conformation can be inferred from the known structure of the complex, and peptides that will show high predicted stability for that conformation will be tested for affinity and specificity.

A final note is warranted about further acceleration of the proposed screening process. The MD stability estimator requires several simulations for each peptide. This may be too computationally demanding if the number of virtual peptides is large. In such cases, the KBP estimator may serve as a "quick and dirty" method for initial screening. The application of the MD estimator could then be restricted to the 20% top-ranking peptides selected by the KBP (Fig. 4) and the final selection would proceed with the combined estimator.

## Methods

### The peptide dataset

This study focused on forty short (10–16 residues) β-hairpin peptides that belong to four families: BHKE/MBH [14,15]; Tryptophan Zipper – TrpZip [16]; bhpW [17,18]; and EPO [19] (Table 1). The developers of these families used diverse design strategies to enforce the β-hairpin structures: Disulfide bridges between the terminals constrain the bhpW and EPO peptides; TrpZip peptides are characterized by strong hydrophobic interactions of four tryptophan residues; and the BHKE hairpin conformation is stabilized by high β-propensity residues and by electrostatic interactions. The NMR structures of eight of these peptides are available. The structures of the other 32 peptides were modeled (using the Swiss-PdbViewer [39] "mutate" tool) based on the NMR structure with the highest sequence similarity. The experimentally measured structural stabilities of these peptides are available in the literature [14-19].

### MD Simulations protocol

All simulations were performed using the molecular modeling package MOIL [40] under the AMBER/OPLS forcefield [41,42] and with TIP3P [43] explicit water model. Peptides were first soaked within a 28 × 28 × 28 Å water box, and clashes were removed by 500 steps of conjugate gradient minimization [44]. Next, the MD simulation started with 30 picoseconds (ps) of heating from 0°K to the designated temperature. This heating stage was followed by 2970 ps of constant temperature simulation. Throughout the MD simulations periodic boundary conditions were applied and the peptide center of mass was constrained to the center of the simulation box. MD parameters were: (a) one-femtosecond time steps; (b) velocity scaling every 30 ps; (c) non-bonded neighbors list update every 20 steps; (d) truncation cutoffs of Van der Waals and electrostatic interactions at 6Å and 8Å, respectively and (e) saving of coordinates every 2ps. For each peptide, 10 simulations were performed with the same initial structure, either an NMR structure or a template-based model, but with different random initial velocities.

The persistence of the initial structures during the simulations was quantified by the root mean square deviation of all the peptide atoms from the initial structure (RMSD_all-atoms_). However, because the trajectories of the RMSD_all-atoms _tend to be rather diverse and noisy, we used the smoother median trajectory (Figure 5). Thus, the structural stability of a peptide was estimated to be proportional to the fraction of the simulation time in which the median trajectory was below a 2.6Å threshold. The selection of this particular threshold is described in the results section.

### Knowledge-Based Potential

The second orthogonal technique we used to estimate structural stability of peptides is motivated by the assumption that local residue preferences have a large impact on the structural stability [45]. The Knowledge-Based Potential estimates the statistical likelihood of finding a given polypeptide in a specific set of {*φ*, *ψ*} torsion angles:

(1) *E*(*residue type*, *φ*, *ψ*) = -log [*P*(*φ*, *ψ *| *type*)]

(2) $$E_{TOTAL}=\sum_{all\;residues}E(residue\;type,\phi ,\psi )$$

Where *P *is the frequency of finding a certain {*φ*, *ψ*} conformation among the occurrences of residue *type *in a large database, *E*(*residue type*, *φ*, *ψ*) is the energy associated with a single residue and *E*_TOTAL _is the total energy of the entire peptide or protein. The parameters of the potential were derived from a set of 1145 solved protein structures from the ASTRAL database (release 1.63) [46]. Since the energy calculation has to be performed only once per peptide, the structural stability is fast to compute. Peptides are predicted to be structurally more stable as their energy decreases.

### Bootstrapping

In order to test the sensitivity of the correlation results to small subsets, we performed bootstrapping analyses with 1000 random samples [53] for each peptide family.

### Data analysis

All the statistical analyses were performed using the statistical R software [54]. Specifically, Pearson's correlations and their p-values (the probability of an error when the null hypothesis of zero correlation is rejected) were calculated using the cor and cor.test functions. Bootstrapping was performed using the replicate and sample functions, and for each bootstrapping sample the correlation and p-value were calculated separately.

## Authors' contributions

KN did all the preliminary studies, designed and performed the Molecular Dynamics simulations and did all the analysis. NK developed and performed the knowledge-based potential calculations. CK supervised the project. All authors contributed to writing the paper, have read and approved the final version of this manuscript.

## References

[B1] Janecka A, Zubrzycka M, Janecki T (2001). Somatostatin analogs. J Pept Res.

[B2] Ueno H, Yamaguchi H, Kangawa K, Nakazato M (2005). Ghrelin: A gastric peptide that regulates food intake and energy homeostasis. Regul Pept.

[B3] Hruby VJ, Agnes RS, Davis P, Ma SW, Lee YS, Vanderah TW, Lai J, Porreca F (2003). Design of novel peptide ligands which have opioid agonist activity and CCK antagonist activity for the treatment of pain. Life Sci.

[B4] Schally AV, Varga JL (1999). Antagonistic analogs of growth hormone-releasing hormone: New potential antitumor agents. Trends Endocrinol Metab.

[B5] Walensky LD, Kung AL, Escher I, Malia TJ, Barbuto S, Wright RD, Wagner G, Verdine GL, Korsmeyer SJ (2004). Activation of Apoptosis in Vivo by a Hydrocarbon-Stapled BH3 Helix. Science.

[B6] Ladner RC (1995). Constrained peptides as binding entities. Trends Biotechnol.

[B7] Li P, Roller PP (2002). Cyclization strategies in peptide derived drug design. Curr Top Med Chem.

[B8] Humphrey JM, Chamberlin AR (1997). Chemical Synthesis of Natural Product Peptides: Coupling Methods for the Incorporation of Noncoded Amino Acids into Peptides. Chem Rev.

[B9] Sidhu S (2001). Phage display in pharmaceutical biotechnology. Curr Opin Biotechnol.

[B10] Wrighton NC, Farrell FX, Chang R, Kashyap AK, Barbone FP, Mulcahy LS (1996). Small peptides as potent mimics of the protein hormone erythropoietin. Science.

[B11] Hann M, Green R (1999). The in-silico world of virtual libraries. Curr Opin Chem Biol.

[B12] Thomas A, Deshayes S, Decaffmeyer M, Van Eyck MH, Charloteaux B, Brasseur R (2006). Prediction of peptide structure: how far are we?. Proteins.

[B13] Sergio M, Ignasi B, Xavier L, Ernest G (2005). Design of enhanced agonists through the use of a new virtual screening method: Application to peptides that bind class I major histocompatibility complex (MHC) molecules. Protein Science.

[B14] Ramírez-Alvarado M, Blanco FJ, Serrano L (2001). Elongation of the BH8 b-hairpin peptide: Electrostatic interactions in β-hairpin formation and stability. Protein Science.

[B15] Pastor MT, Lopez de la Paz M, Lacroix E, Serrano L, Perez-Paya E (2002). Combinatorial approaches: A new tool to search for highly structured β-hairpin peptides. Proc Natl Acad Sci USA.

[B16] Cochran AG, Skelton NJ, Starovasnik MA (2001). Tryptophan zippers: Stable, monomeric β-hairpins. Proc Natl Acad Sci USA.

[B17] Russell SJ, Blandl T, Skelton NJ, Cochran AG (2003). Stability of Cyclic, β-Hairpins: Asymmetric Contributions from Side Chains of a Hydrogen-Bonded Cross-Strand ResiduePair. J Am Chem Soc.

[B18] Cochran AG, Tong RT, Starovasnik MA, Park EJ, McDowell RS, Theaker JE, Skelton NJ (2001). A minimal peptide scaffold for beta-turn display: optimizing a strand position in disulfide-cyclized beta-hairpins. J Am Chem Soc.

[B19] Skelton NJ, Russell S, de Sauvage F, Cochran AG (2002). Amino Acid Determinants of b-Hairpin Conformation in Erythropoeitin Receptor Agonist Peptides Derived from a Phage Display Library. J Mol Biol.

[B20] Zanuy D, Ma B, Nussinov R (2003). Short peptide amyloid organization: stabilities and conformations of the islet amyloid peptide NFGAIL. Biophys J.

[B21] Zanuy D, Nussinov R (2003). The sequence dependence of fiber organization: a comparative molecular dynamics study of the Islet amyloid polypeptide segments 22–27 and 22–29. J Mol Biol.

[B22] Zanuy D, Porat Y, Gazit E, Nussinov R (2004). Peptide sequence and amyloid formation: molecular simulations and experimental study of a human Islet amyloid polypeptide fragment and its analogs. Structure.

[B23] Tsai H, Zanuy D, Haspel N, Gunasekaran K, Ma B, Tsai CJ, Nussinov R (2004). The stability and dynamic of the human Calcitonin amyloid peptide DFNKF. Biophys J.

[B24] Pande VS, Rokhsar DS (1999). Molecular dynamics simulations of unfolding and refolding of a β-hairpin fragment of protein G. Proc Natl Acad Sci USA.

[B25] Galzitskaya OV, Higo J, Finkelstein AV (2002). Alpha-helix and beta-hairpin Folding from experiment, analytical theory and molecular dynamics simulations. Curr Protein Pept Sci.

[B26] Simmerling C, Strockbine B, Roitberg AE (2002). All-atom structure prediction and folding simulations of a stable protein. J Am Chem Soc.

[B27] Levitt M, Sharon R (1988). Accurate simulation of protein dynamics in solution. Proc Natl Acad Sci.

[B28] Tsai J, Gerstein M, Levitt M (1997). Simulating the minimum core for hydrophobic collapse in globular proteins. Protein Science.

[B29] Raschke TM, Tsai J, Levitt M (2001). Quantification of the hydrophobic interaction by simulations of the aggregation of small hydrophobic solutes in water. Proc Natl Acad Sci.

[B30] Bond CJ, Wong KB, Clarke J, Fersht AR, Daggett V (1997). Characterization of residual structure in the thermally denatured state of barnase by simulation and experiment: description of the folding pathway. Proc Natl Acad Sci USA.

[B31] Tsai J, Levitt M, Baker D (1999). Hierarchy of structure loss in MD simulations of src SH3 domain unfolding. J Mol Biol.

[B32] Lu H, Skolnick J (2001). A distance-dependent atomic knowledge-based potential for improved protein structure selection. Proteins.

[B33] Mayor U, Guydosh NR, Johnson CM, Grossmann JG, Sato S, Jas GS, Freund SM, Alonso DO, Daggett V, Fersht AR (2003). The complete folding pathway of a protein from nanoseconds to microseconds. Nature.

[B34] Bystroff C, Simons KT, Han KF, Baker D (1996). Local sequence-structure correlations in proteins. Curr Opin Biotechnol.

[B35] Shortle D (2002). Composites of local structure propensities: Evidence for local encoding of long-range structure. Protein Sci.

[B36] Wodak S, Rooman M (1993). Generating and testing protein folds. Curr Opin Struct Biol.

[B37] Fujitsuka Y, Chikenji G, Takada S (2006). SimFold energy function for de novo protein structure prediction: consensus with Rosetta. Proteins.

[B38] Rohl CA, Strauss CE, Misura KM, Baker D (2004). Protein structure prediction using Rosetta. Methods Enzymol.

[B39] Guex N, Peitsch MC (1997). SWISS-MODEL and the Swiss-PdbViewer: An environment for comparative protein modeling. Electrophoresis.

[B40] Elber R, Roitberg A, Simmerling C, Goldstein R, Li H, Verkhiver G, Keasar C, Zhang J, Ulitsky A (1994). MOIL: A program for simulation of macromolecules. Comp Phys Comm.

[B41] Weiner SJ, Kollman DA, Case UC, Ghio C, Alagona G, Profeta S, Weiner P (1984). A new force field for molecular mechanical simulation of nucleic acids and proteins. J Amer Chem Soc.

[B42] Jorgensen WL, Tirado-Rives J (1988). The OPLS Potential Functions for Proteins. Energy Minimizations for Crystals of Cyclic Peptides and Crambin. J Am Chem Soc.

[B43] Jorgensen WL, Chandrasekhar J, Madura JD (1983). Comparison of simple potential functions for simulating liquid water. J Chem Phys.

[B44] Powell MJD (1977). Restart procedures of the conjugate gradient method. Mathematical Programming.

[B45] Ramachandran GN, Sasisekharan V (1968). Conformation of polypeptides and proteins. Adv Protein Chem.

[B46] Brenner SE, Koehl P, Levitt M (2000). The ASTRAL compendium for sequence and structure analysis. Nucleic Acids Research.

[B47] Mackerell AD, Feig M, Brooks CL (2004). Extending the treatment of backbone energetics in protein force fields: Limitations of gas-phase quantum mechanics in reproducing protein conformational distributions in molecular dynamics simulations. Journal of Computational Chemistry.

[B48] Mohanty D, Dominy BN, Kolinski A, Brooks CL, Skolnick J (1999). Correlation between knowledge-based and detailed atomic potentials: application to the unfolding of the GCN4 leucine zipper. Proteins.

[B49] Bystroff C, Garde S (2003). Helix propensities of short peptides: molecular dynamics versus bioinformatics. Proteins.

[B50] Berman HM, Westbrook L, Feng Z, Gilliland G, Bhat TN, Shindyalov IN, Bourne PE (2002). The Protein Data Bank. Nucleic Acids Research.

[B51] Eisenberg D, McLachlan AD (1986). Solvation energy in protein folding and binding. Nature.

[B52] Lazaridis T, Karplus M (1999). Heat capacity and compactness of denatured proteins. Biophysical Chemistry.

[B53] Efron B, Tibshirani R (1993). An introduction to the Bootstrap.

[B54] Development R, Core Team R A Language and Environment for Statistical Computing. http://www.R-project.org.

